# The mediating role of rumination in the relationship between insomnia and non-suicidal self-injury of college students

**DOI:** 10.3389/fpsyg.2024.1504890

**Published:** 2024-12-16

**Authors:** Hanqing Wang, Hongli Sun

**Affiliations:** ^1^Training and Research Center of Ideological and Political Work Team of Ministry of Education (Shaanxi Normal University), Xi'an, China; ^2^Psychological Health Education and Counseling Center, Sanda University, Shanghai, China

**Keywords:** insomnia, college students, non-suicidal self-injury, rumination, mediating role

## Abstract

**Introduction:**

Non-suicidal self-injury (NSSI) is a high-risk factor for suicide, which is widespread among college students and is closely associated with psychological issues. Insomnia tends to cause mental instability, and chronic insomnia can trigger severe mood swings, including anxiety, depression, and irritability. It can also lead to memory loss and metabolic disorders. College students are in a critical period of self-development, with significant vulnerability to insomnia and NSSI. This study investigated the influence of insomnia on NSSI among college students and examined the significance of rumination as a mediating factor.

**Methods:**

Using random cluster sampling, we selected 667 college students from Jiangsu, China, and evaluated them using the Athens Insomnia Scale (AIS), Adolescent Self-Injury Scale (ASIS), and Ruminative Responses Scale (RRS). The significance of the mediating effect of rumination on the relationship between insomnia and NSSI among college students was tested using Bootstrap methods.

**Results:**

The fact that the students were only children was statistically significant for rumination scores (*p* < 0.05). The results showed a significant positive correlation among insomnia, NSSI, and rumination (*r* = 0.198, 0.737, 0.243, respectively; *p* < 0.001). Insomnia of college students affects NSSI directly and indirectly through rumination.

**Conclusion:**

Rumination fully mediated the relationship between insomnia and NSSI among college students, indicating an indirect influence of insomnia on NSSI. Total effect value is 0.192. Rumination plays a complete mediating role in the relationship between insomnia and NSSI among college students, with a mediating effect of 84.21%.

## 1 Introduction

Non-suicidal self-injury (NSSI) refers to the intentional harming of body tissues without suicidal intent, encompassing more than a dozen different manifestations, such as cutting, scratching, burning, pulling out hair, and hurting oneself with sharp objects (Nock, 2010; Zhong et al., 2018a). According to a survey conducted among 223 adolescents receiving care at a community clinic for mood disorders and self-injurious behaviors, approximately two-thirds reported experiencing severe nightly sleep disturbances (McGlinchey et al., 2017). It has become a major public health issue among college students and has gained increasing attention.

NSSI among college students is influenced by several external factors and internal conflicts, and the consequences of NSSI bring indelible harm to individuals and their families (Whitlock et al., 2006). A report published in 2002 investigated the occurrence of NSSI among a school-based cohort of adolescents and showed that the prevalence of “self-injurious” behavior was approximately 14% (Ross and Heath, 2002). A previous comprehensive meta-analysis revealed that the lifetime prevalence of self-injurious behavior among global adolescents was about 22.1% between 1989 and 2018 (Lim et al., 2019). Globally, NSSI is the third leading contributor to disability-adjusted life years (DALYs) among adolescents aged 10–24 years (Voss et al., 2020). A review has estimated an alarmingly high lifetime prevalence of NSSI among Chinese youth, reaching a staggering 24.7% (Qu et al., 2023). Non-suicidal NSSI is frequently comorbid with depression and eating disorders and also leads to an increased risk of suicidal ideation and suicidal behavior (Groschwitz et al., 2015; Xu H. et al., 2022). Self-injurious behavior significantly increases the risk of suicide, with a comorbidity rate between self-harm and suicide reaching 7.7% (Voss et al., 2020; Zhong et al., 2018b). Considering the universality and harmfulness of NSSI, this study aims to investigate the influencing factors and psychological mechanisms underlying NSSI.

### 1.1 Insomnia and non-suicidal self-injury behavior

Insomnia refers to the subjective experience of dissatisfaction with the quality and duration of sleep, which affects social functioning despite having a suitable sleep environment and opportunities (Xu et al., 2022a). A previous meta-analysis revealed that the pooled prevalence of insomnia in the general population was 22.0% (Zeng et al., 2020). With the development of society, the pressure of study and life of college students has increased, leading to increasingly serious sleep problems, among which the incidence of insomnia is increasing. Consequently, it leads to short-term or long-term health issues such as physical fatigue, decline in physical function, cognitive impairment, obesity, and depression (Jiang et al., 2015; Xu et al., 2022b). According to the cognitive model of insomnia, adolescents with insomnia have lower functional emotional responses and inhibition of impulse control, leading to the implementation of NSSI (Harvey, 2002). A meta-analysis indicates that sleep disturbances serve as a significant risk factor for suicidal ideation and suicide attempts among healthy adolescents. The results emphasize that adolescents with sleep disturbances are more inclined to attempt suicide and experience suicidal ideation than controls (Baldini et al., 2024; Williams et al., 2020). Research has demonstrated a close relationship between insomnia and NSSI, highlighting the significance of investigating sleep issues to prevent NSSI (Latina et al., 2021).

### 1.2 The mediating role of rumination

Rumination occurs when an individual focuses excessively on negative emotions, thoughts, or behaviors, repeatedly reflects on their causes and consequences, and experiences feelings of being blocked and unable to actively address problems (Nolen-Hoeksema, 1991). Current empirical research utilizing longitudinal follow-up methods to explore the relationship between rumination and sleep quality indicates that rumination promotes cognitive arousal and influences individual sleep quality (Woods and Scott, 2016 Kroese et al., 2016). According to the 3-P model of insomnia (Dhabhar et al., 1997), maintenance factors, such as incorrect sleep cognition, synergistically contribute to the development of persistent insomnia. Studies have demonstrated a significant negative correlation between rumination and sleep quality (Baglioni et al., 2010). When an individual cannot solve a problem due to persistent negative thinking, the non-adaptive characteristics can abruptly increase psychological pressure and foster inclinations toward self-harm (Bandel and Brausch, 2020). Intense rumination can cause an escalating accumulation of negative emotions, easily triggering NSSI (Selby et al., 2013). The emotional cascade model suggests that rumination increases negative emotions, such as those caused by sleep deprivation, which in turn intensifies rumination, thus creating a vicious cycle known as the “emotional cascade.” Moreover, NSSI allows individuals to momentarily divert their attention from rumination to intense physical sensations, thereby disrupting the emotional cascade process (Selby et al., 2013). College students might use NSSI as a direct and effective means to alleviate negative emotions, such as sleep deprivation.

This study combined the 3-P model of insomnia and the emotional cascade model to construct a mediation model to examine the mechanisms underlying the relationship between insomnia and NSSI among college students. Based on relevant theoretical and empirical evidence, the following hypotheses were proposed:

*H*: Insomnia is positively associated with NSSI, and rumination mediates the relationship between insomnia and NSSI among college students.

## 2 Materials and methods

### 2.1 Participants

A total of 680 college students from two universities in Nanjing, China, were selected for this study. The stratified cluster sampling method was employed to select 40 classes, with 10 classes drawn from each grade level, spanning freshmen to seniors. From each selected class, half of the students were randomly chosen to participate in the experiment. The study was designed as a double-blind experiment and conducted individually by a single researcher, who ensured that all tests were administered only after obtaining informed consent from the participants. A total of 680 questionnaires were distributed, of which 667 (98.09%) were effectively collected. Among the respondents, 426 (63.87%) were male students and 241 (36.13%) were female. The student population comprised 362 urban students (54.27%) and 305 rural students (45.73%). In addition, 402 subjects (60.27%) were only children, and 265 (39.73%) were not. The ages of the subjects ranged from 17 to 24 years, with an average age of (19.81 ± 1.31) years.

### 2.2 Measures

#### 2.2.1 Athens Insomnia Scale

The Athens Insomnia Scale (AIS) proposed by Professor Dan Sedmark from the College of Medicine at Ohio State University in the United States was adopted (Soldatos et al., 2003; Soldatos et al., 2000). The scale consisted of 8 questions with 4 response options: no problem, slight influence, significant influence, and no sleep or severe influence. Scores were accumulated, with a total score of less than 4 indicating no sleep disorder, between 4 and 6 suggesting possible insomnia, and 6 or more indicating the presence of insomnia. This scale is a widely used self-report scale for assessing insomnia symptoms in Chinese individuals (Zhang et al., 2021), and the Cronbach’s alpha value for the entire scale was 0.846.

#### 2.2.2 Adolescent Self-Injury Scale

The Adolescent Self-Injury Scale (ASIS) revised by Feng Yu and complied by Zheng Ying (Zheng, 2006; Feng, 2008), comprising a total of 19 questions, was adopted in this study. Among these questions, 18 were dedicated to exploring the frequency and severity of 18 different types of NSSI, and the rest were open-ended. The NSSI score was calculated as the product of the frequency and severity scores. The measurement outcomes demonstrated excellent internal consistency in this study (Cronbach’s α = 0.912).

#### 2.2.3 Ruminative Responses Scale

Rumination was assessed using the Ruminative Response Scale (RSS) developed by Professor Susan Nolen-Hoeksema and translated into Chinese by Yang Juan (Nolen-Hoeksema and Davis, 1999; Yang et al., 2009). The scale comprised 21 questions encompassing depression-related factors, reflective factors, and compulsive meditation factors. A higher total score corresponded to a higher level of rumination. The measurement outcomes demonstrated excellent internal consistency in this study (Cronbach’s α = 0.966).

### 2.3 Procedure

The study was reviewed and approved by the Ethics Committee of Sanda University (No. 2023006). The inclusion criteria were college students from freshman year to senior year, with those having suicidal tendencies excluded. All participants willingly consented to participate and provided their informed consent, which included the study’s objectives, provisions for anonymity, and the confidentiality and security of data. The inclusion criteria for the study subjects were college students enrolled in the two selected universities, excluding those with suicidal tendencies. Each questionnaire took approximately 10 min to complete, and the data were collected from January to March 2024.

To minimize the effect of common method variance (CMV) on the results, unrotated exploratory factor analysis was conducted using the Harman factor test on the 59 items encompassing three scales. The results showed that 7 factors had eigenvalues greater than 1. The first factor explained 33.60% of the total variance, which is less than 40%, indicating that the questionnaire data did not suffer from severe CMV (Podsakoff et al., 2003).

### 2.4 Data analysis

The data were analyzed using SPSS 26.0. All questionnaires were filled out anonymously. The data were used for *t*-tests, correlation analysis, descriptive statistics, etc. The mediation effects of insomnia in the relationship between social exclusion and NSSI were examined using Hayes’ Process 4.3 plug-in Model 4, supplemented with Bootstrap analysis.

## 3 Results

### 3.1 Statistical analysis of demographic differences in insomnia, NSSI, and rumination

Demographic variables for insomnia, NSSI, and rumination among college students were analyzed using the independent samples *t*-test. The results showed that the students were only children were statistically significant for rumination scores (*p* < 0.05). However, place of birth were not statistically correlated with insomnia, NSSI, or rumination scores. Detailed results are shown in Table 1.

**Table 1:** Differences in demographic characteristics of insomnia, NSSI, and rumination among college students (*x*¯ ± *s*).

Demographic variables	Options	Insomnia	*t*	NSSI	*t*	Rumination	*t*
Gender	Male	5.89 ± 4.35	−0.96	1.68 ± 5.70	−1.31	40.09 ± 14.17	−1.34
	Female	6.24 ± 4.59		2.69 ± 11.27		41.60 ± 13.54	
Origin of student	Town	5.73 ± 4.43	−1.85	2.27 ± 7.26	0.76	39.74 ± 14.77	−1.83
	Rural area	6.36 ± 4.43		1.78 ± 9.14		41.70 ± 12.86	
Only child or not	Yes	5.98 ± 4.30	0.25	1.85 ± 8.27	0.78	40.63 ± 12.64	0.02*
	No	6.07 ± 4.65		2.35 ± 8.02		40.65 ± 15.76	

*p < 0.05, **p < 0.01, ***p < 0.001, the same below.

### 3.2 Descriptive statistics and correlation analysis

Descriptive statistics and correlation analysis were conducted on insomnia, NSSI, and rumination among college students. The results, shown in Table 2, indicate significant positive correlations between these variables. Specifically, there is a significant positive correlation between insomnia and NSSI (*r* = 0.20, *p* < 0.001), insomnia and rumination (*r* = 0.74, *p* < 0.001), and NSSI and rumination (*r* = 0.24, *p* < 0.001). Detailed results are provided in Table 2.

**Table 2:** Correlation matrix (r) of Insomnia, NSSI and rumination among college students.

	*x̄* ± *s*	Insomnia	NSSI	Rumination
Insomnia	5.96 ± 4.46	1		
NSSI	2.02 ± 8.13	0.20^***^	1	
Rumination	40.44 ± 14.00	0.74^***^	0.24^***^	1

### 3.3 Mediation analysis of insomnia, NSSI, and rumination in college students

The mediation effect was analyzed using model 4 in Hayes’ macro program with insomnia as the independent variable, NSSI as the dependent variable, rumination as the mediator variable, and gender and age as the control variable. The results of the mediation effect analysis showed that the 95% confidence interval (CI) for each path did not include 0, indicating that the mediation effect of the sense of rumination on insomnia and NSSI was significant. The results showed a significant positive correlation between insomnia and NSSI (*β* = 0.19, *t* = 5.07, *p* < 0.001) among college students, indicating that the path coefficient of insomnia and NSSI was significant in the direct effect model. However, after adding rumination as the mediator variable, the positive correlation between insomnia and NSSI was no longer significant (*β* = 0.04, *t* = 0.64, *p* > 0.05), indicating an absence of significant mediation effects in the model. Therefore, rumination plays a full mediation role in the relationship between insomnia and NSSI among college students. Detailed results are shown in Table 3.

**Table 3:** The mediation effect analysis of rumination.

Outcome	Predictors	*R*	*R*-sq	*F* (df)	*β*	*t*
NSSI	Insomnia	0.22	0.05	11.53^***^ (665)	0.19	5.07^***^
	Gender				0.10	1.30
	Age				−0.07	−2.29^*^
Rumination	Insomnia	0.74	0.54	263.31^***^ (665)	0.74	28.03^***^
	Gender				0.05	0.96
	Age				0.01	0.62
NSSI	Insomnia	0.26	0.07	12.49^***^ (664)	0.04	0.64
	Rumination				0.21	3.83^***^
	Gender				0.09	1.17
	Age				−0.07	−2.41^*^

**Figure 1:**
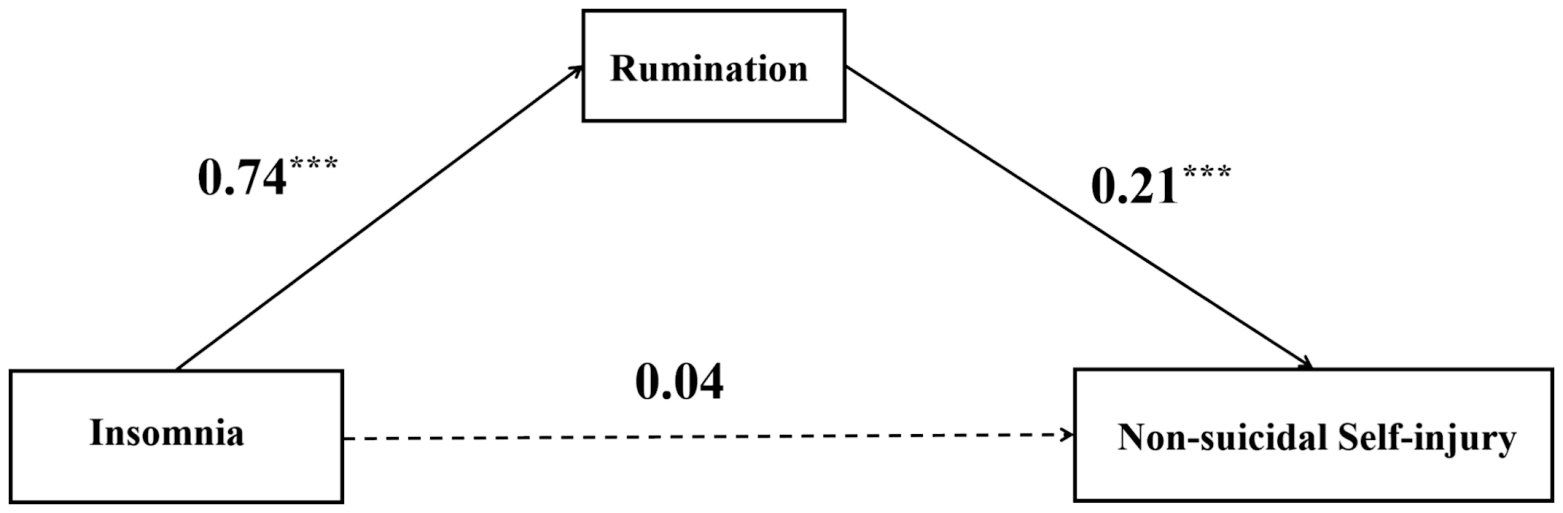
The full mediation effect model. Note: ***p <0.001.

The mediating effect of rumination between insomnia and NSSI among college students was examined by Bootstrap analysis with 5,000 replicate samples, and the results are shown in Table 4. The direct effect of insomnia on NSSI was significant, with an effect value of 0.04. The Bootstrap 95% confidence intervals (upper and lower limits) did not encompass 0 for any of the paths, suggesting a mediating effect of rumination between insomnia and NSSI among college students. This finding supports Hypothesis 2 with an effect value of 0.16. Moreover, the proportion of the mediating effect to the total effect is 84.21%, and the model diagram of the mediating effect is detailed in Figure 1.

**Table 4:** Bootstrap analysis for full mediation effects.

Paths	Value	SE	ULCI	LLCI
Total effect	0.19	0.04	0.27	0.12
Insomnia → NSSI	0.04	0.06	0.15	−0.07
Insomnia→ Rumination → NSSI	0.16	0.07	0.29	0.02

## 4 Discussion

This study examined how insomnia impacts the NSSI among college students and investigated the mediating role of rumination. It was found that rumination played a mediating role between insomnia and NSSI.

The detection rate of insomnia (scale score greater than 6 points) among college students was 37.33%, and the detection rate of suspicious insomnia (scale score between 3 and 6 points) was 38.23%, consistent with the results of a previous study (Cao et al., 2017). In addition, approximately 23.83% of college students reported experiencing NSSI for at least one time, consistent with the research results of 24.7% obtained by Qu et al. (2023). This result could be attributed to the fact that women have richer emotional experiences, greater sensitivity, and are more susceptible to negative emotions in their academic and personal lives, leading to higher levels of psychological distress and a greater tendency to resort to NSSI (Gandhi et al., 2015).

### 4.1 The relationship between insomnia, NSSI, and rumination of college students

Insomnia of college students shows a significant positive correlation with NSSI, i.e., the more serious the insomnia of college students, the more serious the NSSI, aligning with previous research (Latina et al., 2021). College students without sleep disorders are physically and mentally happy, less physically impaired, and more energized for daytime activities (Mehta, 2022). Insomnia may affect the clearing of traumatic memories in individuals, prolonging the impact of traumatic memories (Chunhua et al., 2019). Moreover, insomnia may increase the probability of NSSI by intensifying feelings of distress, hopelessness, and individual impulsiveness (Bauducco et al., 2019). Insomnia in college students has a significant correlation with rumination. According to the Cognitive Model of Insomnia (Harvey, 2002), insomniacs tend to worry excessively about sleep and its consequences, and such negative cognitions induce autonomic arousal and emotional distress, with recurrent thoughts about the causes of negative emotional experiences and undesirable outcomes leading to high levels of rumination. Notably, rumination has a significant positive correlation with NSSI, i.e., the higher the level of rumination indicates a more serious NSSI, which is consistent with the previous research results (Nagy et al., 2023). According to the reaction style theory, rumination reduces social support and problem-solving ability, thereby strengthening the influence of negative factors. Intense rumination among adolescents can lead to decreased self-control levels, ultimately resulting in NSSI (Miranda et al., 2013). Previous studies have shown that adolescents can be treated with cognitive behavioral therapy for insomnia (Crevits et al., 2024).

### 4.2 The mediating role of rumination between insomnia and NSSI among college students

Rumination plays a complete mediating role in the relationship between insomnia and NSSI among college students, with a mediating effect of 84.21%, indicating that insomnia of college students affects NSSI directly and indirectly through rumination. This result validates the emotional cascade model to a certain extent, which suggests that the cascade of rumination and negative emotions creates aversive states, and the NSSI disperses this cascades, thereby reducing the occurrence of negative emotions (Selby et al., 2013). Negative emotions can induce cognitive biases in individuals when confronted with stressful life events related to sleep problems, leading them to excessively focus on and negatively evaluate potential factors contributing to poor sleep quality, ultimately degrading the sleep quality of college students and leading to insomnia.

According to the insomnia maintenance cognitive model, prolonged sleep delay may lead individuals to worry excessively about subsequent sleep quality, triggering and prolonging non-adaptive stress-sleep interactions (Harvey, 2002). Long-term sleep deficiency or insomnia can impair the ability of individuals to regulate negative emotions (Niu and Snyder, 2023), while rumination is considered a maladaptive coping strategy and a unique cognitive mechanism. This mechanism is related to the increase in negative emotions and the decrease in inhibition of negative information. When facing negative emotions reinforced by rumination, students have difficulty initiating and sustaining positive emotion regulation efforts, which aggravates pain and makes NSSI more inclined to avoid aversive experiences (Selby et al., 2013). For college students with NSSI, psychological interventions can incorporate methods such as cognitive behavioral therapy for insomnia and stimulus control therapy to improve sleep conditions, thereby addressing NSSI.

### 4.3 Limitations and future directions

This study employs a cross-sectional design to explore the relationship between insomnia, NSSI, and rumination in college students. The limitation of a cross-sectional study is that it restricts the consideration of insomnia as a static factor for exploring the impact on NSSI and rumination. In the future, the mechanism between insomnia and NSSI will be investigated through a longitudinal follow-up study. The questionnaire assessment method used in this study did not incorporate psychiatric diagnosis, and relevant diagnostic studies will be considered. Finally, this study is a single-center study, only considering Jiangsu Province. Therefore, multi-center large-scale random cluster sampling will be taken into account. Our future study aims to mitigate the occurrence of NSSI among college students by seeking interpersonal support, establishing positive behavioral patterns, and reducing negative cognitive-emotional regulation. This approach will effectively reduce the comorbidity of NSSI and suicide and effectively intervene in the occurrence of NSSI among college students.

## 5 Conclusion

In conclusion, this study utilized a questionnaire survey to investigate the mediating effect of rumination on the relationship between insomnia and NSSI. The results of mediation effect analysis showed that insomnia had a significant direct effect on NSSI, but after adding the mediator variable rumination, insomnia had no significant direct effect on NSSI, indicating that rumination played a fully mediating role between insomnia and NSSI. Students with good sleep quality find it easier to relieve fatigue, enhance immune function, promote development, possess greater vitality for interpersonal communication, a stable state of mind, and an optimistic attitude, thus reducing the generation of negative emotions (Xie et al., 2023). Good emotional and cognitive experience are crucial in mitigating the risk of NSSI. To effectively prevent the physical and psychological harm to college students caused by NSSI and reduce the probability of college suicides, schools and families should urge college students to develop reasonable work and rest habits, thereby reducing the number and frequency of staying up late.

## Data Availability

The original contributions of this study are outlined in the article and supplementary materials. For further inquiries, please contact the corresponding author.
