# Supplementary material for: The mediating role of rumination in the relationship between insomnia and non-suicidal self-injury of college students
Source: Front Psychol. 2024 Dec 16;15:1504890. doi: 10.3389/fpsyg.2024.1504890 (PMC11682892; doi:10.3389/fpsyg.2024.1504890)
Supplement: Supplementary file 1 [file Table_1.DOCX]

**The mediating role of Rumination in the relationship between Insomnia and Non-suicidal Self-injury of College Students**

**Adolescent Self-Injury Scale**

we are doing a survey on the relationship between insomnia, self-injury behavior and learning burnout among college students. Your cooperation is very important to us, and we hope to obtain your support. The results are for data analysis only, and your answers will never be leaked, please rest assured. Thank you for your answers!

Step 1: Gender

Men and women

2. Grade

Freshman, sophomore, junior, senior, other

3. Your major

Arts Science and Engineering (music, art, physical education, etc.) Medical Science

Step 4: Age

5. Long-term residence of the family

Rural town

6. Whether you are an only child

Yes or no

7. Whether to serve as a student leader in school

Yes or no

8. Family financial situation

Better is generally worse

9. Father's education level

Junior high school or technical college or above (including junior college)

10. Mother's education level

Junior high school or technical college or above (including junior college)

11. Monthly household income

<5000 5000-10000 10000-15000 >15000

12. Family structure

Parents sound divorced single orphans other

13. Family education model

Democratic Pal indulgence coddling authority and strict neglect of indifference

14. Primary caregiver during childhood

Parents (foreign) Grandfather, mother other

(Self-injurious behavior)

The number of times an action has occurred in your past life and the extent to which it has harmed your body

0 times 1 time 2-4

5 times or more without mild moderate severe extremely severe

1. Intentionally using glass, knife, etc., to cut their own skin;

2. Deliberately poking the wound to prevent it from healing;

3. Intentionally burning/scalding your own skin with a cigarette butt, lighter or other object;

4. Deliberately tattooing characters or patterns on the body (except for tattooing);

5. Deliberately scraping and bleeding your own skin;

6. Deliberately stabbing something into the skin or under the nail;

7. Bruising by intentionally hitting your head against something;

8. Deliberately pulling out your own hair;

9. Intentionally hitting hard objects such as walls or glass with their hands;

10. Scratching oneself deliberately and violently to the point of bruising or bleeding;

11. Intentionally pricking a part of the body with a needle, nail, or other object to bleed blood;

12. Deliberately rubbing the skin to bleed;

13. Intentionally punching yourself so that you bruise;

14. Deliberately strangle your wrist or other parts with a rope or other objects;

15. Intentionally causing others to hit or bite you in order to harm your own body;

16. Intentionally electrocuting yourself in a non-life-threatening situation

17. Bite yourself on purpose to break your skin;

18. Intentionally lighting a fire or touching a flame in your hand;

If there are any other ways you intentionally hurt yourself that are not mentioned in this questionnaire, please write:

Frequency of occurrence

Degree of physical injury

0 times 1 time 2-4 times 5 times or more

No mild moderate severe extremely severe

1. Intentionally using glass, knife, etc., to cut their own skin;

0 times 1 time 2-4 times 5 times or more

(to the extent of bodily harm) intentionally cutting your own skin with a glass, knife, etc

No mild moderate severe extremely severe

2. Deliberately poking the wound to prevent it from healing;

0 times 1 time 2-4 times 5 times or more

Intentionally poking open a wound (to the extent of bodily harm) to prevent healing;

No mild moderate severe extremely severe

3. Intentionally burning/scalding your own skin with a cigarette butt, lighter or other object;

0 times 1 time 2-4 times 5 times or more

(to the extent of bodily harm) intentionally burning/scalding your own skin with a cigarette butt, lighter or other object;

No mild moderate severe extremely severe

4. Deliberately tattooing characters or patterns on the body (except for tattooing);

0 times 1 time 2-4 times 5 times or more

(degree of bodily harm) intentional tattooing of words or designs on the body (except for tattooing purposes);

No mild moderate severe extremely severe

5. Deliberately scraping and bleeding your own skin;

0 times 1 time 2-4 times 5 times or more

(to the extent of bodily harm) deliberately scraping one's own skin to bleed;

No mild moderate severe extremely severe

6. Deliberately stabbing something into the skin or under the nail;

0 times 1 time 2-4 times 5 times or more

(to the extent of bodily harm) deliberately stabbing something into the skin or under a nail

No mild moderate severe extremely severe

7. Bruising by intentionally hitting your head against something;

0 times 1 time 2-4 times 5 times or more

(to the extent of bodily injury) intentionally hitting something with the head so as to cause bruising

No mild moderate severe extremely severe

8. Deliberately pulling out your own hair;

0 times 1 time 2-4 times 5 times or more

(to the extent of bodily harm) deliberately pulling out one's own hair

No mild moderate severe extremely severe

9. Intentionally hitting hard objects such as walls or glass with their hands;

0 times 1 time 2-4 times 5 times or more

Intentionally hitting something hard, such as a wall or glass with your hand

No mild moderate severe extremely severe

10. Scratching oneself deliberately and violently to the point of bruising or bleeding;

0 times 1 time 2-4 times 5 times or more

To intentionally and violently scratch oneself (to the point of being bruised or bleeding)

No mild moderate severe extremely severe

11. Intentionally pricking a part of the body with a needle, nail, or other object to bleed blood;

0 times 1 time 2-4 times 5 times or more

(to the extent of bodily injury) deliberately pricking a part of the body with a needle, nail, or other object to bleed

No mild moderate severe extremely severe

12. Deliberately rubbing the skin to bleed;

0 times 1 time 2-4 times 5 times or more

Deliberately rubbing the skin to bleed (to the extent of bodily harm)

No mild moderate severe extremely severe

13. Intentionally punching yourself so that you bruise;

0 times 1 time 2-4 times 5 times or more

(to the extent of physical injury) deliberately punching oneself so that bruising occurs

No mild moderate severe extremely severe

14. Deliberately strangle your wrist or other parts with a rope or other objects;

0 times 1 time 2-4 times 5 times or more

(to the extent of bodily harm) deliberately strangling oneself by the wrist, etc., with a rope or other object

No mild moderate severe extremely severe

15. Intentionally causing others to hit or bite you in order to harm your own body;

0 times 1 time 2-4 times 5 times or more

(to the extent of physical harm) intentionally causing someone to hit or bite you in order to harm your own body

No mild moderate severe extremely severe

16. Intentionally electrocuting yourself in a non-life-threatening situation

0 times 1 time 2-4 times 5 times or more

Intentionally electrocuting oneself in a non-life-threatening situation

No mild moderate severe extremely severe

17. Bite yourself on purpose to break your skin;

0 times 1 time 2-4 times 5 times or more

Bite yourself intentionally so that your skin is broken

No mild moderate severe extremely severe

18. Intentionally lighting a fire or touching a flame in your hand;

0 times 1 time 2-4 times 5 times or more

(to the extent of bodily harm) intentionally lighting a fire or touching a flame in one's hand

No mild moderate severe extremely severe
